# Activated Histone Acetyltransferase p300/CBP-Related Signalling Pathways Mediate Up-Regulation of NADPH Oxidase, Inflammation, and Fibrosis in Diabetic Kidney

**DOI:** 10.3390/antiox10091356

**Published:** 2021-08-26

**Authors:** Alexandra-Gela Lazar, Mihaela-Loredana Vlad, Adrian Manea, Maya Simionescu, Simona-Adriana Manea

**Affiliations:** Institute of Cellular Biology and Pathology, “Nicolae Simionescu” of the Romanian Academy, 050568 Bucharest, Romania; alexandra.lazar@icbp.ro (A.-G.L.); loredana.antonescu@icbp.ro (M.-L.V.); adrian.manea@icbp.ro (A.M.); maya.simionescu@icbp.ro (M.S.)

**Keywords:** diabetes, nephropathy, epigenetics, histone acetylation, p300/CBP, NADPH oxidase, oxidative stress

## Abstract

Accumulating evidence implicates the histone acetylation-based epigenetic mechanisms in the pathoetiology of diabetes-associated micro-/macrovascular complications. Diabetic kidney disease (DKD) is a progressive chronic inflammatory microvascular disorder ultimately leading to glomerulosclerosis and kidney failure. We hypothesized that histone acetyltransferase p300/CBP may be involved in mediating diabetes-accelerated renal damage. In this study, we aimed at investigating the potential role of p300/CBP in the up-regulation of renal NADPH oxidase (Nox), reactive oxygen species (ROS) production, inflammation, and fibrosis in diabetic mice. Diabetic C57BL/6J mice were randomized to receive 10 mg/kg C646, a selective p300/CBP inhibitor, or its vehicle for 4 weeks. We found that in the kidney of C646-treated diabetic mice, the level of H3K27ac, an epigenetic mark of active gene expression, was significantly reduced. Pharmacological inhibition of p300/CBP significantly down-regulated the diabetes-induced enhanced expression of Nox subtypes, pro-inflammatory, and pro-fibrotic molecules in the kidney of mice, and the glomerular ROS overproduction. Our study provides evidence that the activation of p300/CBP enhances ROS production, potentially generated by up-regulated Nox, inflammation, and the production of extracellular matrix proteins in the diabetic kidney. The data suggest that p300/CBP-pharmacological inhibitors may be attractive tools to modulate diabetes-associated pathological processes to efficiently reduce the burden of DKD.

## 1. Introduction

Diabetic kidney disease (DKD), the major microvascular complication of both type I and type II diabetes, is a complex multifactorial renal disorder having a detrimental impact on the patient’s quality of life and life-span expectation [1,2,3]. Dysfunction of glomerular endothelial cells and podocytes, thickening of the glomerular basement membrane, mesangial cells hypertrophy and proliferation, progressive accumulation of mesangial extracellular matrix (ECM) components, podocyte damage, and disruption of glomerular endothelium fenestrations are the main structural alterations ultimately leading to glomerulosclerosis; a pathological condition that is further responsible for increased intraglomerular capillary pressure, hyperfiltration, and eventually kidney failure [4,5,6].

Regardless of major achievements in DKD therapeutics, the current pharmacological strategies that include blood glucose, lipids, and blood pressure control can only delay the progression of renal damage. Consequently, dialysis, and ultimately kidney transplantation, remain the main therapeutic options for kidney failure in end-stage DKD [7,8]. Thus, the development of additional or supportive, glomerular cell-oriented pharmacological interventions acting in conjunction with standard anti-hyperglycaemic and anti-hypertensive drugs may contribute to formulating novel treatment algorithms to efficiently reduce the burden of DKD than is currently possible.

Hyperglycaemia, the main metabolic abnormality in diabetes, induces glomerular structural-functional dysfunction via multiple mechanisms that broadly include the formation of advanced glycation end products (AGEs), overproduction of detrimental reactive oxygen species (ROS), hemodynamic alterations, metabolic dysfunction, inflammation, phenotypic changes of the glomerular cells, excess synthesis, and accumulation of ECM components [7,9,10,11].

Studies on human diabetic kidney biopsies and experimental models of diabetes provide reliable evidence that ROS overproduction, typically driven by the up-regulated NADPH oxidase (Nox) family, are the major triggers of the proinflammatory and profibrotic signalling pathways [12,13,14,15,16,17,18]. Noteworthy, hyperglycaemia-induced metabolic memory via Nox-enhanced ROS production provides a possible explanation of the limitations of the current glucose-lowering therapeutic strategies in diabetic patients [19]. Consequently, Nox subtypes, and their upstream regulators, may become important candidates as pharmacological targets in DKD.

Accumulating evidence demonstrates that the dysregulation of epigenetic mechanisms, namely, DNA methylation, post-translational modifications of histones, and non-coding RNA, plays a major role in the pathology of cardiovascular disorders (CVD), and potentially in DKD [20,21,22,23]. Among other epigenetic modifiers, histone acetylation-based mechanisms have emerged as attractive therapeutic targets in experimental CVD. Lysine-specific acetylation of nucleosomal histones is tightly regulated by the coordinated activities of two major enzymatic systems, namely, histone acetyltransferases (HAT) and histone deacetylases (HDAC). Canonically, HAT-mediated histone acetylation induces chromatin relaxation due to electric charge neutralization of the histone tails, a condition that enables DNA-transcription factors interactions and activation of gene expression. Other than histones, several HAT subtypes regulate the function of a range of transcription factors to induce or repress the gene expression [24].

Abnormal expression levels of both HAT and HDAC subtypes and lysine-specific histone acetylation patterns have been mechanistically implicated in an increasing number of human and experimental models of CVD. Histone acetyltransferase p300/CBP is a ubiquitously expressed transcriptional co-activator and regulates the expression of genes by several interconnected mechanisms comprising histone acetylation-induced chromatin relaxation owing to the intrinsic HAT activity of p300, recruitment of RNA polymerase II, and also serves as a scaffold protein to form active transcriptional complexes within gene promoter and enhancer regions. Moreover, p300 directly regulates the function of several transcription factors (e.g., NF-kB) to enhance the expression of the target genes [25,26,27].

Histone acetyltransferase p300/CBP is an important transcriptional co-activator regulating the expression of oxidative stress- and inflammation-related genes. Previously, we have demonstrated a mechanistic connection among activated histone-acetylation pathways and Nox-derived ROS overproduction in experimental models of diabetes and atherosclerosis [28,29,30]. Yet, the role of p300/CBP in DKD remains elusive. Based on the important functions of the dysregulated histone acetylation-related mechanisms in vascular pathology, we hypothesized that in DKD, p300/CBP may act as an upstream regulator of Nox expression, ROS production, inflammation, and fibrosis. We provide here evidence that activation of p300/CBP mediates the up-regulation of Nox subtypes, ROS overproduction, and increases the expression of proinflammatory molecules and extracellular matrix proteins in the mouse diabetic kidney.

Our data propose p300/CBP-pharmacological inhibitors as attractive tools to modulate molecular and cellular processes mechanistically linked to the pathology of DKD. 

## 2. Materials and Methods

### 2.1. Materials

Unless specifically mentioned, standard chemicals and reagents were obtained from Sigma-Aldrich (Darmstadt, Germany). C646 {4-[4-[[5-(4,5-Dimethyl-2-nitrophenyl)-2-furanyl]methylene]-4,5-dihydro-3-methyl-5-oxo-1H-pyrazol-1-yl] benzoic acid}, a cell-permeable, highly selective, reversible inhibitor of histone acetyltransferase p300/CBP (purity ≥ 98%, high-performance liquid chromatography analysis) was obtained from Sigma-Aldrich/Calbiochem (Darmstadt, Germany). Primary and secondary antibodies were from Santa Cruz Biotechnology (Heidelberg, Germany), Thermo Fisher Scientific (Vienna, Austria), Abcam (Cambridge, UK), and Diagenode (Seraing, Belgium). The pNF-κB-Luc (#219078), pC/EBP-Luc (#240112), pGAS-Luc (#219091), and pISRE-Luc (#219089) *cis*-reporter plasmids were purchased from Stratagene (La Jolla, San Diego, CA, USA). The American Type Culture Collection (ATCC) (Manassas, VA, USA) derived human embryonic kidney 293 (HEK293) cell line was used. 

### 2.2. Procedure for Induction of Experimental Diabetes and Treatment Strategy in Mice

Male C57BL/6J (Stock No: 000664) mice obtained from The Jackson Laboratory were used. The mice reproduced in our SPF animal facility, were exposed to 12-h of light/dark cycles and had access to a standard rodent chow diet and water ad libitum. At 8 weeks of age, the mice were intraperitoneally (i.p.) injected with 55 mg/kg streptozotocin (STZ), for 5 successive days to induce experimental diabetes [9,31,32]. Age-matched C57BL/6J mice injected with citrate buffer (pH 4.5) were employed as non-diabetic controls. The installation of hyperglycaemia in the diabetic animal group was confirmed by measuring the glucose level in the blood collected by tail puncture, one week after the last STZ injection. The animals were further randomized into three experimental groups to receive (i.p.) 10 mg/kg C646 or its vehicle (5% DMSO + 95% PBS, pH 7.4), every other day for 4 weeks: (i) non-diabetic mice + vehicle (*n* = 10/group), (ii) diabetic mice + vehicle (*n* = 10/group), and (iii) diabetic mice + C646 (*n* = 10/group). The chosen dose and the procedure of C646 administration to mice were in good agreement with previous reports [33,34]. At the end of the treatment procedure, the animals were sacrificed and the blood was collected by cardiac puncture. After perfusion of mice with calcium-containing PBS (pH 7.4), the kidneys were surgically harvested. The animal studies were conducted in accordance with the guidelines of EU Directive 2010/63/EU and the experimental protocols were approved by the ethical committee of the Institute of Cellular Biology and Pathology “Nicolae Simionescu” (#11/29.06.2016, #384/09.02.2018, #6/07.04.2021).

### 2.3. Quantitative Real-Time Polymerase Chain Reaction (Real Time-PCR) Assay 

The kidneys were suspended in RNase inhibitor-containing lysis buffer (Sigma) and subjected to glass bead-based (1.0 mm diameter) tissue homogenization (BioSpec, Bartlesville, OK, USA). Total RNA was extracted from the tissue homogenates using a column-based purification kit (Sigma). The synthesis of complementary DNA (cDNA) was performed by reverse transcribing 0.5 μg of total RNA using the MMLV reverse transcriptase enzyme (Thermo Fisher). The relative mRNA transcript levels corresponding to specific Nox subtypes, inflammatory, and fibrotic markers were determined by amplification of the cDNA employing a real-time PCR machine (LightCycler ^TM^ 480 II, Roche, Basel, Switzerland) and quantified by the C_T_ comparative method [35]. The β-actin mRNA expression level was used for internal normalization. The oligonucleotide primer sequences are shown in Table S1 in the Supplementary Materials file. 

### 2.4. Western Blot Assay

Kidneys derived from each animal were suspended in RIPA lysis buffer containing a protease inhibitor cocktail (Sigma) and subjected to homogenization as indicated above. Protein denaturation was performed in Laemmli electrophoresis sample buffer (Serva) at 95 °C for 20 min. Protein samples (30 μg/lane) were separated by SDS-PAGE and transferred onto nitrocellulose membranes (Bio-Rad). The membranes were incubated for 12 h (4 °C) with primary antibodies against H3K27ac (rabbit polyclonal, C15410174, dilution 1:1000), H3pan (mouse monoclonal, C15200011, dilution 1:1000), Nox1 (rabbit polyclonal, ab131088, concentration 0.5 µg/mL), Nox2 (rabbit polyclonal, PA5-79118, concentration 0.5 µg/mL), Nox4 (rabbit polyclonal, sc-30141, 1:200), or β-actin (mouse monoclonal, sc-47778, 1:500) followed by one-hour exposure (room temperature) to anti-rabbit IgG-HRP (sc-2370, 1:2000) or anti-mouse IgG-HRP (sc-2031, dilution 1:2000) secondary antibodies. Protein bands were detected by chemiluminescence imaging (ImageQuant LAS 4000 system, Fujifilm, Tokyo, Japan). TotalLab ^TM^ (Newcastle upon Tyne, UK)-based densitometric analysis of the protein levels was carried out using the expression level of β-actin protein as internal normalization. 

### 2.5. Histochemistry and Immunofluorescence Microscopy

After harvesting, the kidneys were fixed overnight at 4 °C in paraformaldehyde 4% solution in phosphate buffer 0.1 M, pH 7.4, cryoprotected in 5%, 10%, 20%, and 50% glycerol solutions in phosphate buffer 0.1 M, pH 7.4, and embedded in optimal cutting temperature compound. Kidney cryosections (5 μm-thick) were mounted onto SuperFrost Plus ^TM^ microscope slides (Thermo Scientific) and stained with hematoxylin-eosin solution or subjected to fluorescence immunolabeling for Nox1 (rabbit polyclonal, ab131088, dilution 1:250), Nox2 (rabbit polyclonal, PA5-79118, dilution 1:250), Nox4 (rabbit polyclonal, sc-30141, dilution 1:50), collagen IV (rabbit polyclonal, ab6586, dilution 1:80), fibronectin (rabbit polyclonal, ab2413, dilution 1:250) or laminin (rabbit polyclonal, PA5-16287, dilution 1:250). As a secondary antibody, Alexa Fluor ^TM^ 594 goat anti-rabbit IgG (A11037, dilution 1:500) was used. In these experiments, 4′,6-diamidino-2-phenylindole (DAPI) stain was used to detect the cell nuclei in the specimens. Sections were examined and photographed with an inverted fluorescence microscope (Zeiss Axio Observer). Quantitative analysis of the staining was carried out using ImageJ software (NIH Image, Bethesda, MD, USA).

### 2.6. Detection of ROS In Situ

The redox-sensitive probe, dihydroethidium (DHE)/hydroethidine, was used for in situ detection of ROS on fresh-frozen 5 μm-thick kidney cryosections as previously described [17]. Briefly, the sections were rinsed for 30 min in PBS (pH 7.4) at room temperature (to remove the OCT) and then incubated with 5 μM DHE in PBS (pH 7.4) at 37 °C for 30 min in a dark humidified chamber slide. After washing in PBS (pH 7.4) to remove the residual DHE, the fluorescence images were taken with an inverted fluorescence microscope (Zeiss Axio Observer). Quantitative analysis of the DHE staining was performed employing ImageJ software (NIH Image, USA).

### 2.7. Luciferase Reporter Assay

HEK293 cells were employed as the luciferase expression system due to their high efficiency of transfection. Twenty-four hours prior to transfection, HEK293 cells were seeded at 1 × 10^5^ cells/well into 12-well tissue culture plates. The Superfect ^TM^ reagent was used for transient transfection of the cells in accordance with the manufacturer’s protocol (Qiagen). The enhancer element configuration and sequence of the *cis*-reporter plasmids were as follows: pNF-κB-Luc [(NF-κB (5×), (TGGGGACTTTCCGC)_5_], pC/EBP-Luc [C/EBP (3×), (ATTGCGCAAT)_3_], pGAS-Luc [GAS (4×), (AGTTTCATATTACTCTAAATC)_4_], and pISRE-Luc [ISRE (5×), (TAGTTTCACTTTCCC)_5_]. The optimized plasmid concentrations were: 0.9 μg/mL of *cis*-reporter plasmid and 0.1 μg/mL pSV-β-galactosidase expression vector (Promega, Walldorf, Germany). The transcription factor/luciferase activity was calculated from the ratio of firefly luciferase (luciferase assay system/Promega) to β-galactosidase level (o-nitrophenyl-β-D-galactopyranoside-based β-galactosidase enzyme assay/Promega). 

### 2.8. Statistical Analysis

Data obtained from at least three independent experiments were expressed as the mean ± standard deviation. Statistical analysis was performed by two-tailed *t*-test and one-way analysis of variance (ANOVA) followed by Tukey’s post hoc test; *p* < 0.05 was considered statistically significant.

## 3. Results

### 3.1. In Diabetic Mice Long-Term Pharmacological Inhibition of p300/CBP Has No Effect on Plasma Level of Glucose and Body Weight

**Figure 1:**
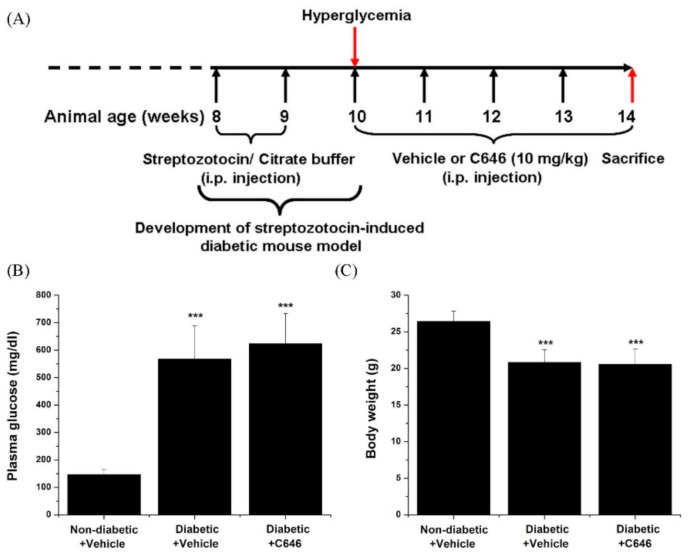
(**A**) Schematic depiction of the experimental set-up to induce diabetes in C57BL/6J mice and the duration of C646/vehicle administration to mice. (**B**,**C**) Plasma glucose levels and body weights were assessed for each animal group at the end of the treatment procedure. *n* = 5–7, *** *p* < 0.001. *p*-values were taken in relation to non-diabetic + vehicle condition.

STZ-induced diabetic C57BL/6J mice were employed as an experimental model to find out whether p300/CBP has a role in the up-regulation of oxidative stress-, inflammation-, and fibrosis-related gene or protein expression levels in the kidney. As schematically depicted in Figure 1A, the animals (*n* = 30) were distributed into experimental groups to receive either C646 pharmacological inhibitor or its vehicle for 4 weeks. We found that after 4 weeks of diabetes, the plasma glucose level was significantly increased (≈4-fold) and the bodyweight reduced (≈20%) in vehicle-treated diabetic mice as compared with vehicle-treated non-diabetic control counterparts. As compared with vehicle-treated diabetic animals, no significant changes in blood glucose levels and body weights were detected following long-term pharmacological blockade of p300/CBP in diabetic mice (Figure 1B,C).

### 3.2. Histone Acetyltransferase p300/CBP Mediates the Up-Regulation of H3K27ac Level in the Diabetic Kidney

**Figure 2:**
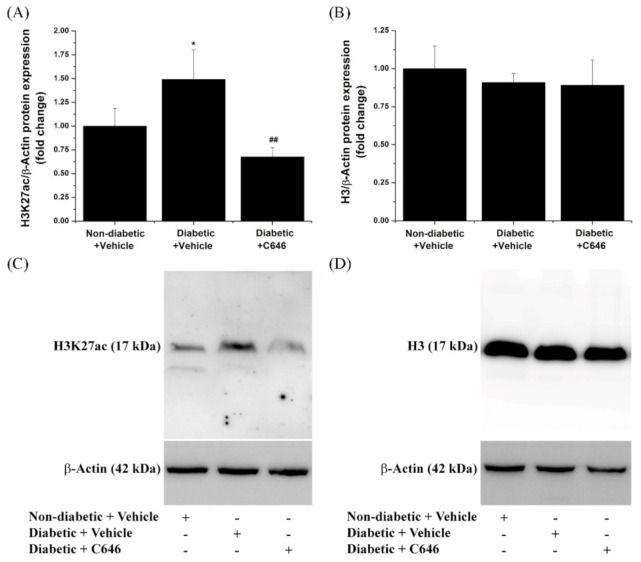
Pharmacological inhibition of histone acetyltransferase p300/CBP suppresses the up-regulation of H3K27ac level in the kidney of diabetic mice. *n* = 3–4, * *p* < 0.05, *p*-value taken in relation to non-diabetic + vehicle condition; ## *p* < 0.01, *p*-value taken in relation to diabetic + vehicle condition. (**A**,**B**) Densitometric analysis of the Western blot data showing the modulation of overall renal H3K27ac and H3 protein expression levels in each animal group. (**C**,**D**) Representative immunoblots depicting the relative expression levels of H3K27ac and H3 in the whole kidney protein extracts.

To investigate the impact of diabetic conditions on p300/CBP-induced histone acetylation in the kidney, we examined the relative level of H3K27ac, an important substrate of p300 biochemical activity and an epigenetic marker of active gene expression [29]. Western blot analysis of the whole kidney protein extracts revealed a significant increase (≈1.5-fold) in H3K27ac levels in the diabetic mice group as compared with non-diabetic animals. Importantly, the diabetes-induced increase in the H3K27ac level was suppressed by 4-week administration of C646 pharmacological inhibitor to diabetic mice. The relative expression of the total H3 protein level in the kidney, analyzed in parallel experiments, remained steady in all experimental groups (Figure 2). 

### 3.3. Diabetes-Activated p300/CBP Signalling Pathways Mediate the Up-Regulation of Renal Nox Expression

**Figure 3:**
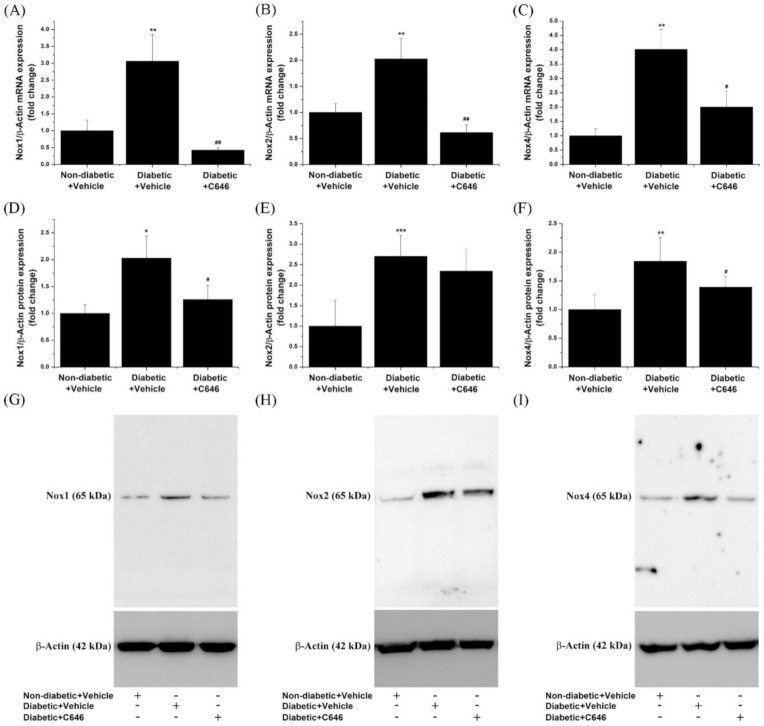
Histone acetyltransferase p300/CBP mediates the up-regulation of Nox expression in the kidney of diabetic mice. (**A**–**C**) Quantitative real-time PCR analysis indicating the suppressive effects of p300/CBP blockade on diabetes-induced enhanced mRNA levels of Nox1, Nox2, and Nox4 subtypes. (**D**–**F**) Densitometric analysis of the Western blot data showing the relative expression levels of Nox1, Nox2, and Nox4 proteins in each experimental condition. (**G**–**I**) Representative immunoblots depicting the modulation of Nox proteins expression. *n* = 3–4, * *p* < 0.05, ** *p* < 0.01, *** *p* < 0.001, *p*-values taken in relation to non-diabetic + vehicle condition; # *p* < 0.05, ## *p* < 0.01, *p*-values taken in relation to diabetic + vehicle condition.

ROS overproduction driven by up-regulated Nox enzymes is acknowledged as the main trigger of oxidative stress-induced renal injury in DKD [12]. To determine whether p300/CBP is implicated in the regulation of the overall Nox expression, the gene and protein expression levels of the Nox1, Nox2, and Nox4 subtypes, the catalytic subunits of the Nox complex, were assessed in the whole kidney extracts. A significant increase in the mRNA and protein levels of Nox1 (mRNA ≈ 3-fold; protein ≈ 2-fold), Nox2 (mRNA ≈ 2-fold; protein ≈ 2.7-fold), and Nox4 (mRNA ≈ 4-fold; protein ≈ 1.85-fold) isoforms were determined in the kidney of diabetic mice after 4 weeks of hyperglycaemia. Long-term blockade of p300/CBP by C646 suppressed the up-regulation of Nox1, Nox2, and Nox4 transcript levels in the diabetic kidney. Furthermore, the protein expression levels of Nox1 and Nox4 subtypes, except the Nox2 isoform, were significantly diminished in response to C646 administration to diabetic mice as compared to non-treated diabetic animals (Figure 3).

**Figure 4:**
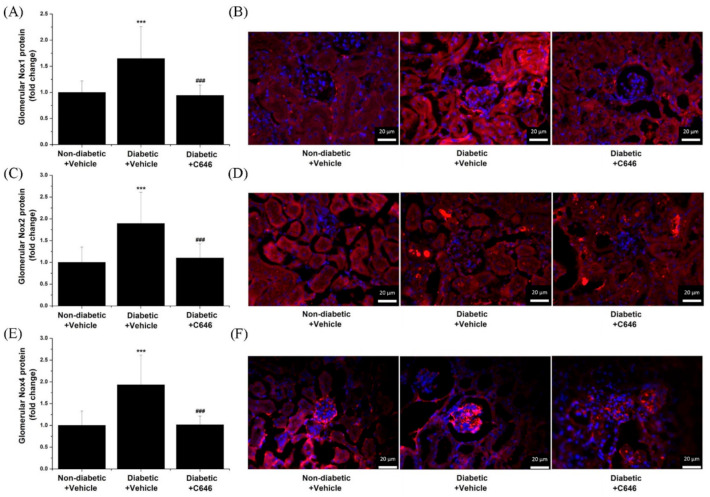
Pharmacological inhibition of histone acetyltransferase p300/CBP by C646 mitigates the glomerular expression of Nox subtypes in diabetic mice. (**A**,**C**,**E**) Quantification of fluorescence immunolabeling for glomerular Nox1, Nox2, and Nox4 subtypes. (**B**,**D**,**F**) Representative IF microscopy images taken at 40× magnification depicting the immunofluorescence staining (red) for Nox subtypes. Sections were counterstained with DAPI (blue) stain to detect the cell nuclei in the specimens. Note that the up-regulation of glomerular Nox1, Nox2, and Nox4 protein levels in diabetic mice is blunted following C646 treatment. *n* = 14–19/condition quantified glomeruli, *** *p* < 0.001, *p*-values taken in relation to non-diabetic + vehicle condition; ### *p* < 0.001, *p*-values taken in relation to diabetic + vehicle condition.

Previous comprehensive studies provided compelling evidence that the up-regulation of Nox enzymes is a key pathogenic mechanism associated with glomerulosclerosis [14,15]. Hitherto, the role of p300/CBP in the regulation of glomerular Nox expression in diabetic conditions remains elusive. Thus, we further examined the relative expression of Nox catalytic subunits in the glomeruli by immunofluorescence (IF) microscopy. Compared to controls (non-diabetic animals), enhanced glomerular immunolabeling of Nox1 (≈1.65-fold), Nox2 (≈1.9-fold), and Nox4 (≈1.95-fold) proteins was detected in diabetic mice. Blockade of p300/CBP resulted in a significant decrease in Nox1, Nox2, and Nox4 protein levels in the renal glomeruli of diabetic mice. Noteworthy, there was an apparent increase in Nox4 immunodetection, suggesting that the Nox4 subtype, rather than Nox1 and Nox2, is abundant in the glomeruli. These data are consistent and extend a previous study demonstrating that Nox4-containing Nox, rather than Nox1 or Nox2, is a major source of ROS in the glomeruli and contributes to oxidative stress-induced renal damage in experimental diabetes [15]. However, the superior antibody-recognition performance of Nox4 protein as well as the accessibility of the antibody to its specific epitopes in IF microscopy assays should be considered (Figure 4).

**Figure 5:**
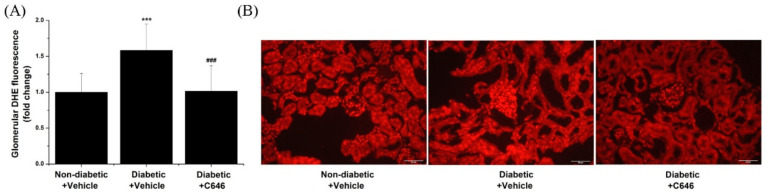
Histone acetyltransferase p300/CBP-dependent induction of in situ glomerular production of ROS in diabetic mice. (**A**) Quantification of glomerular intensity of ROS-induced DHE specific fluorescence signal. (**B**) Representative fluorescence microscopy images (40× magnification) depicting the down-regulatory effects of p300/CBP pharmacological blockade on glomerular ROS formation. *n* = 17–21/condition quantified glomeruli, *** *p* < 0.001, *p*-values taken in relation to non-diabetic + vehicle condition; ### *p* < 0.001, *p*-values taken in relation to diabetic + vehicle condition.

To further examine the possible association of Nox upregulation with ROS overproduction, the glomerular formation of ROS was assessed by in situ labelling employing the DHE redox-sensitive probe. The results showed that pharmacological blockade of p300/CBP significantly reduced the glomerular ROS production in diabetic mice as compared with vehicle-treated diabetic mice (Figure 5).

### 3.4. Pharmacological Inhibition of p300/CBP Suppresses the Diabetes-Induced Enhanced Expression of Pro-Inflammatory and Pro-Fibrotic Molecules in the Kidney

Multiple pathogenic mechanisms, including Nox-activated redox-sensitive signalling pathways, leading to augmented expression of pro-inflammatory and pro-fibrotic mediators, have been shown to promote and accelerate renal injury in DKD [16,17,18]. Thus, our next query was to uncover the function of histone acetyltransferase p300/CBP as a co-transcriptional regulator of both inflammation- and fibrosis-related genes in the diabetic kidney. To this purpose, we examined the mRNA levels of selective molecules mediating glomerular monocyte/macrophage infiltration and inflammation, as well as ECM components whose enhanced accumulation contributes to glomerulosclerosis in diabetes. 

**Figure 6:**
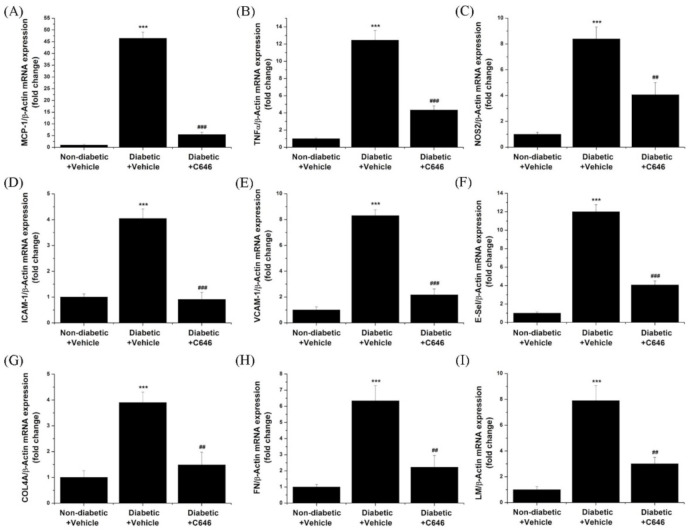
Quantitative real-time PCR analysis indicating the down-regulatory effects of C646-induced p300/CBP inhibition on diabetes-induced augmented mRNA levels of (**A**) MCP-1, (**B**) TNFα, (**C**) NOS2, (**D**) ICAM-1, (**E**) VCAM-1, (**F**) E-selectin, (**G**) collagen IV/COL4A, (**H**) fibronectin/FN, and (**I**) laminin/LM. *n* = 3–4, *** *p* < 0.001, *p*-values taken in relation to vehicle-treated non-diabetic mice condition; ## *p* < 0.01, ### *p* < 0.001, *p*-values taken in relation to vehicle-treated diabetic mice condition.

Compared to controls (non-diabetic animals), whole kidney gene expression analysis demonstrated a significant increase in mRNA levels of pro-inflammatory mediators. These are: monocyte chemotactic protein 1/MCP-1 (≈45-fold), tumour necrosis factor α/TNFα (≈12-fold), nitric oxide synthase 2/NOS2 (≈8-fold), intercellular adhesion molecule 1/ICAM-1 (≈4-fold), vascular cell adhesion molecule 1/VCAM-1 (≈8-fold), E-selectin (≈12-fold). In addition, significant augmented mRNA levels of pro-fibrotic mediators [collagen IV (≈4-fold), fibronectin (≈6-fold), and laminin (≈8-fold)] was detected. Interestingly, the latter diabetes-induced up-regulatory gene expression effects were significantly diminished in C646-treated diabetic mice (Figure 6).

### 3.5. Pharmacological Inhibition of p300/CBP Attenuates the Activity of NF-kB and STAT Transcription Factors

**Figure 7:**
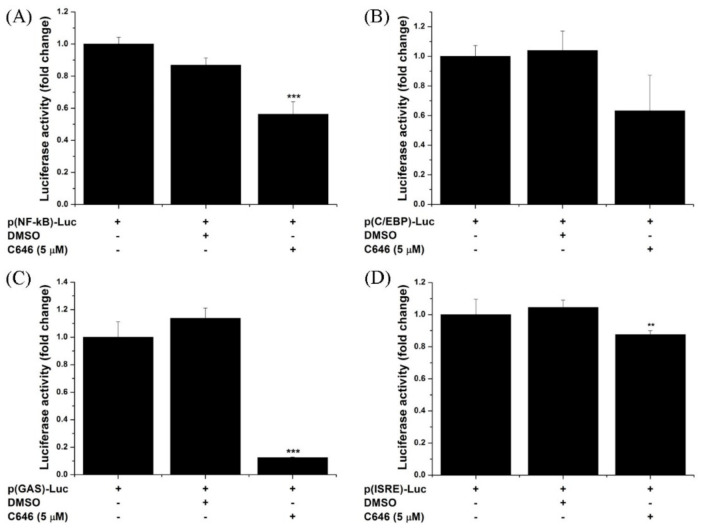
P300/CBP-dependent regulation of the luciferase level directed by the activation of (**A**) NF-kB, (**B**) C/EBP, (**C**) GAS, and (**D**) ISRE enhancer elements in HEK293 cells transfected with the corresponding transcription factor *cis*-reporter plasmids. *n* = 3–6, ** *p* < 0.01, *** *p* < 0.001, *p*-values taken in relation to vehicle (DMSO)-treated cells condition.

Reportedly, p300 plays a key role in regulating the expression of pro-inflammatory molecules by modulating the function of the NF-kB transcription factor [36,37]. Hence, we questioned the involvement of other transcription factors known to regulate both Nox subtypes and selective diabetes-relevant pro-inflammatory and pro-fibrotic molecules [38,39,40,41,42,43]. To this purpose, we analyzed the role of p300/CBP in mediating C/EBP and STAT transcription factor activities employing transient transfection of HEK293 reporter cells, with pNF-κB-Luc, pC/EBP-Luc, pGAS-Luc, and pISRE-Luc *cis*-reporter plasmids containing highly conserved NF-kB, C/EBP, GAS, or ISRE *cis*-acting elements. Twenty-four hours after transfection, the cells were exposed for an additional 24 h to 5 μM C646, vehicle (DMSO), or culture medium alone and then subjected to luciferase level detection as described above. The pharmacological blockade of p300/CBP resulted in a significant down-regulation of luciferase level directed by NF-kB, GAS or ISRE elements, except C/EBP. Interestingly, the blockade of p300/CBP virtually abolished the luciferase level directed by GAS elements, the typically bind STAT1/STAT3 heterodimer as compared with ISRE elements that characteristically mediate the transcriptional response of STAT1/STAT2 complex (Figure 7).

### 3.6. Activation of p300/CBP-Related Signalling Pathways Mediates Diabetes-Induced Glomerular Hypertrophy and Accumulation of Extracellular Matrix Proteins

**Figure 8:**
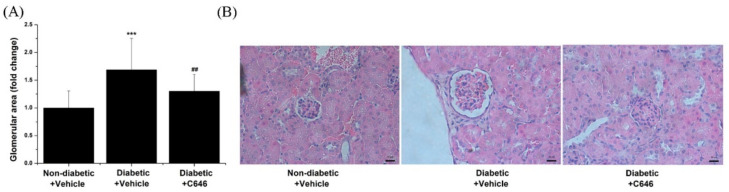
Long-term administration of C646 pharmacological inhibitor of p300/CBP prevents glomerular hypertrophy in diabetic mice. (**A**) Quantification of the relative glomerular area in each experimental animal group. (**B**) Representative hematoxylin-eosin phase-contrast microscopy images taken at 40× magnification depicting the staining of glomeruli. Note that C646 treatment of diabetic mice reduced significantly the relative glomerular hypertrophy to the level of control non-diabetic mice. *n* = 23/condition quantified glomeruli, *** *p* < 0.001, *p*-values taken in relation to non-diabetic + vehicle condition; ## *p* < 0.01, *p*-values taken in relation to diabetic + vehicle condition.

Glomerular hypertrophy typically induced by mesangial cell hypertrophy and proliferation, and accumulation of the ECM proteins is the main structural feature associated with the pathology of DKD. Morphometric analysis of the kidney specimens demonstrated that blockade of p300/CBP significantly reduces diabetes-induced (≈1.7-fold) glomerular hypertrophy (Figure 8).

**Figure 9:**
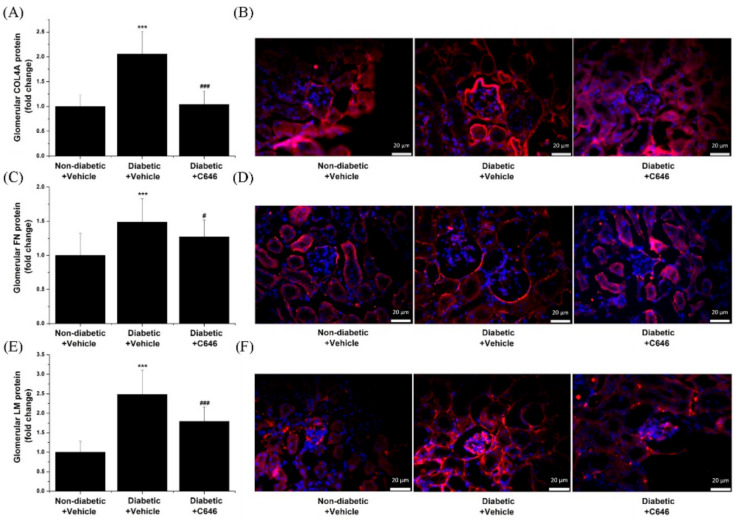
Blockade of histone acetyltransferase p300/CBP reduces the glomerular expression of extracellular matrix proteins collagen IV, fibronectin, and laminin in diabetic mice. (**A**,**C**,**E**) Quantification of fluorescence immunolabeling for glomerular collagen IV/COL4A, fibronectin/FN, and laminin/LM. (**B**,**D**,**F**) Representative immunofluorescence (IF) microscopy images taken at 40× magnification depicting the IF staining (red) for COL4A, FN, and LM. Sections were counterstained with the DAPI stain (blue) to detect the cell nuclei in the specimens. *n* = 18–23/condition quantified glomeruli, *** *p* < 0.001, *p*-values taken in relation to non-diabetic + vehicle condition; # *p* < 0.05, ### *p* < 0.001, *p*-values taken in relation to diabetic + vehicle condition.

Considering the aforementioned findings highlighting that pharmacological blockade of p300/CBP prevents the up-regulation of collagen IV, fibronectin, and laminin transcript levels in the kidney of diabetic mice, we next examined the effect of p300/CBP blockade on the glomerular protein levels. As revealed by IF microscopy, in the diabetic kidney, a significant increase in the glomerular collagen IV/COL4A (≈2-fold), fibronectin/FN (≈1.5-fold), and laminin/LM (≈2.5-fold) was detected. The level of ECM proteins, namely, collagen IV, fibronectin, and laminin was significantly lower in the glomeruli of C646-treated diabetic mice as compared with vehicle-injected diabetic animals (Figure 9). Altogether, these data point to p300/CBP as an important component of a complex transcriptional hub that integrates and transduces signals of diabetic factors to induce renal structural and functional alterations in DKD.

## 4. Discussion

The importance of the complex functional networking among oxidative stress, inflammation, and fibrosis in diabetes-induced renal disorders cannot be overemphasized. Over the past two decades, multiple molecular triggers and signalling pathways of glomerular capillary dysfunction contributing to kidney failure have been determined [1,2,3]. Yet, despite the significant advances in clinical and experimental research in diabetes and its complications, the diagnosis and effective treatment of DKD remains challenging [4,5,6,7,8]. Noteworthy, due to the metabolic memory in diabetes (an emergent and rapidly evolving epigenetic-related mechanistic concept) and in spite of the therapeutic control of hyperglycaemia, diabetes pathological long-lasting effects persist and continue to promote systemic cellular detrimental effects [44,45,46,47]. Consequently, in order to efficiently counteract/attenuate the diabetes-accelerated renal failure, other intrinsic glomerular cell-specific signalling pathways should be considered for drug targeting.

Emerging evidence demonstrates that the diabetes-associated histone acetylation-based epigenetic alterations induce specific transcriptomic instructions in the aorta of mice that regulate important pathological aspects of diabetes-related vascular disorders, including inflammation and oxidative stress [30]. To further uncover potential culprit epigenetic-based pathways underlying renal damage in DKD, we design experiments to find out whether the transcriptional co-activator p300/CBP is mechanistically implicated in the process of oxidative stress, inflammation, and fibrosis in the kidney of diabetic mice.

The novel findings generated by this study on the diabetic kidney disease are: (i) pharmacological inhibition of p300/CBP reduces the level of H3K27ac, an epigenetic mark of active gene expression; (ii) histone acetyltransferase p300/CBP mediates the diabetes-induced mRNA and protein level of Nox subtypes; (iii) blockade of p300/CBP suppresses diabetes-induced glomerular ROS production in situ; (iv) pharmacological inhibition of p300/CBP reduces the transcript levels of pro-inflammatory (MCP-1, TNFα, NOS2, ICAM-1, VCAM-1, E-selectin) and pro-fibrotic molecules (collagen IV, fibronectin, laminin); (v) blockade of p300/CBP reduces the activity of NF-kB and STAT pro-inflammatory transcription factors in vitro; (vi) diabetes-activated p300/CBP-related signalling pathways induce glomerular hypertrophy and an enhanced level of ECM proteins, key pathological features of mesangial expansion leading to kidney failure in diabetes.

Previous comprehensive studies demonstrated that renal Nox subtypes are up-regulated in the human and STZ-induced diabetic mice, a clinically relevant experimental model of DKD and that pharmacological targeting or genetic manipulation of the expression of different Nox subtypes reduces oxidative stress-mediated renal injury in diabetes [15,16]. Yet, other than direct targeting of individual Nox subtype function, reducing the up-regulated Nox expression and the ensuing oxidative stress as well as down-regulating the expression of proinflammatory and profibrotic molecules by selective pharmacological targeting of common up-stream regulators may represent attractive therapeutic strategies in reducing the burden of DKD.

Pharmacological blockade of p300/CBP co-transcriptional activator emerged as a potential way to intervene in several human malignancies [34,48]; however, its possible role in DKD is yet to be determined. Evidence indicates that in addition to histone acetylation-induced chromatin relaxation, p300 activates the function of the NF-kB proinflammatory transcription factor, and consequently may induce the expression of a large number of genes potentially contributing to multiple pathological aspects of CVD such as inflammation and Nox-derived excessive production of ROS [29,37]. Thus, complex co-operative mechanisms involving epigenetic changes in chromatin conformation and lysine-acetylation of transcription factors are likely to be involved in p300-mediated gene transcription. 

Our experiments were intended to uncover new mechanisms responsible for the diabetes-induced renal disease such as the role of p300/CBP in mediating renal oxidative stress, inflammation, and fibrosis. To this purpose, we employed C646, a reversible, cell-permeable pyrazolone-based inhibitor that competes with acetyl-CoA for the histone acetyltransferase p300/CBP-containing Lys-CoA binding pocket. In addition, in accordance with the manufacturer’s specifications (Calbiochem), C646 exhibits high selectivity for p300/CBP activity and less inhibitory effects against other HAT subtypes including aralkylamine *N*-acetyltransferase, p300/CBP-associated factor (PCAF), GCN5, Rtt109, Sas, and MOZ histone acetyltransferases.

Apart from beneficial effects demonstrated in different experimental models of cancer [49], it has been increasingly shown that pharmacological targeting of the up-regulated p300/CBP activity with C646 inhibitor represents a promising way to intervene in the progression of metabolic and cardiovascular disorders. Reportedly, C646 reduced skeletal muscle atrophy in *db/db* mice, an experimental model of type 2 diabetes [50]. In addition, C646-mediated blockade of p300 improved coronary flow in an experimental model of heart failure [33] and reduced cardiac fibrosis in hypertensive mice [51].

Considering the fact that the level of H3K27ac was found significantly elevated in diabetic kidneys, indicative of p300/CBP overactivity, the diabetic mice were subjected to C646 treatment in order to attenuate the p300/CBP-induced renal epigenomic alterations and transcription factor activation potentially contributing to up-regulation of pro-oxidant, pro-inflammatory, and profibrotic genes in response to diabetic insults. Noteworthy, whole-kidney analysis revealed that C646-induced pharmacological inhibition of p300/CBP suppresses the up-regulation of global histone acetylation level, namely, H3K27ac, an epigenetic mark of active gene expression, indicating the implication of p300/CBP in mediating these effects.

Previously, we reported that under pro-inflammatory conditions, p300/CBP mediates the up-regulation of Nox1, Nox2, Nox4, and Nox5 expression in cultured human macrophages [29]. In addition, we demonstrated that the p300 protein physically interacts with Nox1-5 gene promoters at the sites of active transcription and that a high concentration of glucose induces H3K27ac enrichment at the level of Nox1, Nox4, and Nox5 gene promoters correlated with the up-regulation of both mRNA and protein levels in cultured human aortic smooth muscle cells [30]. Considering the fact that p300/CBP mediates the induction of Nox expression in response to pro-inflammatory or diabetic factors, we hypothesized that p300/CBP could become an additional therapeutic target in DKD to attenuate oxidative stress-induced kidney damage. 

Whole renal tissue gene and protein expression analysis revealed that p300/CBP-activated signalling pathways contribute to the overall up-regulation of Nox1, Nox2, and Nox4 mRNA expression and protein levels in the diabetic kidney. Consistent with these findings, pharmacological blockade of p300/CBP suppressed the diabetes-induced glomerular ROS production in situ. Regarding the functional significance of Nox subtypes up-regulation, in a previous comprehensive study, it was demonstrated that the induction of Nox4 rather than Nox1 or Nox2 is implicated in ROS overproduction and oxidative stress-induced kidney failure [15]. Our data are in good agreement and extend this study, and further demonstrate that Nox4, rather than Nox1 and Nox2 subtypes is abundantly expressed in the mouse glomeruli. Yet, as revealed by several in vitro and in vivo studies, the expression of all Nox subtypes and the ensuing ROS overproduction are induced in various cell types in the kidney (i.e., endothelial cells, mesangial cells, podocytes) in response to diabetic conditions by molecular mechanisms that broadly implicate the activation of pro-inflammatory signalling pathways [52,53,54,55,56]. Consistent with this evidence, it was recently demonstrated that ROS overproduction driven by both Nox1 and Nox2 contribute to oxidative stress and kidney injury in diabetic mice [18]. Collectively, the data of our study suggest that the activation of p300/CBP in response to diabetic conditions induces oxidative stress in the kidney via multiple, Nox subtype-dependent, excessive production of ROS. Moreover, pharmacological blockade of p300/CBP significantly reduced the diabetes-associated inflammatory response in the kidney, as demonstrated by the knock-down effects on MCP-1, TNFα, NOS2, ICAM-1, VCAM-1, and E-selectin transcript levels. We also found that in addition to NF-kB, the function of STAT pro-inflammatory transcription factors is reduced in response to p300/CBP inhibition. Noteworthy, NF-kB and STAT are important transcriptional regulators of Nox expression, a condition that may partially explain the down-regulatory effects of p300/CBP pharmacological blockade on Nox1, Nox2, and Nox4 gene expression and protein levels in the diabetic mice kidney [42,43]. This evidence further supports and expands the potential role of p300/CBP as a key modulator of both oxidative stress- and inflammation-related gene expression levels in DKD.

Glomerular hypertrophy generally caused by mesangial cell proliferation and accumulation of ECM proteins is a reliable index of mesangial expansion and subsequent glomerulosclerosis, the main structural abnormality inducing kidney failure in diabetes [1,2,3]. Evidence exists that ROS overproduction, typically generated by activated Nox, and excess production of inflammatory mediators are involved in the mesangial cell phenotypic alterations and increased synthesis of matrix proteins [14]. We provide evidence that in the diabetic kidney, glomeruli enlargement correlates with increased expression of collagen IV, fibronectin, and laminin at both mRNA and protein levels. Importantly, pharmacological inhibition of p300/CBP significantly reduced the diabetes-associated glomerular enlargement and the expression of profibrotic markers of mesangial expansion. Our data are in good agreement and extend a previous study demonstrating that p300/CBP mediates the function of TGFβ1, an important profibrotic factor in mesangial cells [57].

There is evidence that HDAC-dependent epigenetic mechanisms are functionally involved in multiple pathological aspects linked to DKD. It was demonstrated that pharmacological inhibition of HDAC attenuates renal oxidative stress, inflammation, and fibrosis, and improves kidney function in experimental diabetes [58,59,60,61,62,63]. In line with these studies, we have previously reported that HDAC-related signalling pathways mediate the up-regulation of vascular Nox expression and markers of oxidative stress both in vitro and in vivo experimental models of diabetes [30]. Interestingly, the pan-HDAC inhibitor trichostatin A activates the ubiquitin-proteasome pathway to induce p300 protein degradation, thus preventing the formation of transcriptional activation complexes [64]. In sum, these findings could explain the occurrence of similar pharmacological effects driven by HAT or HDAC inhibitors; considering the fact that these enzymatic systems have opposite biochemical functions.

## 5. Conclusions

In conclusion, we provide here evidence that, in the diabetic kidney, the activation of histone acetyltransferase p300/CBP enhances ROS production (most probable generated by the up-regulated Nox), inflammation, and the production of extracellular matrix proteins. Based on the fact that p300/CBP is an important co-transcriptional activator regulating the expression of a wide range of pro-oxidant, pro-inflammatory, and pro-fibrotic genes, we postulate that pharmacological targeting of p300/CBP could become a promising supportive therapeutic option in DKD. Further preclinical and clinical studies should address the relevance of p300/CBP in the pathophysiological context of human DKD in order to translate these findings to human pathology. 

## Data Availability

Data is contained within the article or Supplementary Material.

## Supplementary Materials

The following supporting material is available online at https://www.mdpi.com/article/10.3390/antiox10091356/s1, Table S1: Sequences and GenBank^®^ accession number of oligonucleotide primers used in real-time PCR assays.
