# Supplementary material for: Activated Histone Acetyltransferase p300/CBP-Related Signalling Pathways Mediate Up-Regulation of NADPH Oxidase, Inflammation, and Fibrosis in Diabetic Kidney
Source: Antioxidants (Basel). 2021 Aug 26;10(9):1356. doi: 10.3390/antiox10091356 (PMC8469026; doi:10.3390/antiox10091356)
Supplement: Supplementary file 1 [file antioxidants-10-01356-s001.zip › antioxidants-1329221-supplementary.pdf]

Gene	GeneBank® Accession Number	Sequences of oligonucleotide primers
**Nox1**	NM_172203.2	S: 5'-CATCCAGTCTCCAAACATGACA-3'<br>A: 5'-GCTACAGTGGCAATCACTCCAG-3'
**Nox2**	FJ168469.1	S: 5'-ACTCCTTGGGTCAGCACTGG-3'<br>A: 5'-GTTCCTGTCCAGTTGTCTTCG-3'
**Nox4**	AF276957.1	S: 5'-TGAACTACAGTGAAGATTTCCTTGAAC-3'<br>A: 5'-GACACCCGTCAGACCAGGAA-3'
**MCP-1**	NM_011333.3	S: 5'-CAGCCAGATGCAGTTAACGC-3'<br>A: 5'-GCCTACTCATTGGGATCATCTTG-3'
**TNFα**	NM_013693.3	S: 5'-TCACCCACACCGTCAGCCGATTT-3'<br>A: 5'-CACCCATTCCCTTCACAGAGCAA-3'
**NOS2**	NM_010927.4	S: 5'-CAAGCACCTTGGAAGAGGAG-3'<br>A: 5'-AAGGCCAAACACAGCATACC-3'
**ICAM-1**	NM_010493.3	S: 5'-TGCGTTTTGGAGCTAGCGGACCA-3<br>A: 5'-CGAGGACCATACAGCACGTGCAG-3'
**VCAM-1**	NM_011693.3	S: 5'-CCTCACTTGCAGCACTACGGGCT-3'<br>A: 5'-TTTTCCAATATCCTCAATGACGGG-3'
**E-selectin**	NM_011345.2	S: 5'-CCAATCTGAAACATTCACCGAGT-3'<br>A: 5'-CGAGTCTTTGGTTCGTTGGATG-3'
**COL4A1**	NR_133670.1	S: 5'-ATCTCTGGGGACAACATCCG-3'<br>A: 5'-CATCTCGCTTCTCTCTATGGTG-3'
**Fibronectin**	NM_010233.2	S: 5'-AAGACCATACCTGCCGAATG-3'<br>A: 5'-GAACATGACCGATTTGGACC-3'
**Laminin**	NM_008480.2	S: 5'-TTACGTGGGTGGATTGCCTCAT-3'<br>A: 5'-TGTTCCTGTCCATCACTGTAGG-3'
**β-Actin**	NM_007393.5	S: 5'-CGTGAAAAGATGACCCAGATCA-3'<br>A: 5'-TGGTACGACCAGAGGCATACAG-3'

S, sense; A, antisense

Table S1. Sequences and GenBank® accession number of oligonucleotide primers used in real-time PCR assays.
